# Taurine in sports and exercise

**DOI:** 10.1186/s12970-021-00438-0

**Published:** 2021-05-26

**Authors:** Jennifer A. Kurtz, Trisha A. VanDusseldorp, J. Andrew Doyle, Jeffrey S. Otis

**Affiliations:** 1 Department of Kinesiology and Health, Georgia State University, 125 Decatur Street, Suite 137, Atlanta, GA 30303 USA; 2 Department of Exercise Science and Sport Management, Kennesaw State University, Kennesaw, GA 30144 USA

## Abstract

**Background:**

Taurine has become a popular supplement among athletes attempting to improve performance. While the effectiveness of taurine as an ergogenic aid remains controversial, this paper summarizes the current evidence regarding the efficacy of taurine in aerobic and anaerobic performance, metabolic stress, muscle soreness, and recovery.

**Methods:**

Google Scholar, Web of Science, and MedLine (PubMed) searches were conducted through September 2020. Peer-reviewed studies that investigated taurine as a single ingredient at dosages of < 1 g - 6 g, ranging from 10 to 15 min-to-2 h prior to exercise bout or chronic dose (7 days- 8 weeks) of consumption were included. Articles were excluded if taurine was not the primary or only ingredient in a supplement or food source, not published in peer-reviewed journals, if participants were older than 50 years, articles published before 1999, animal studies, or included participants with health issues. A total of 19 studies met the inclusion criteria for the review.

**Results:**

Key results include improvements in the following: VO_2_max, time to exhaustion (TTE; *n* = 5 articles), 3 or 4 km time-trial (*n* = 2 articles), anaerobic performance (*n* = 7 articles), muscle damage (*n* = 3 articles), peak power (*n* = 2 articles), recovery (*n* = 1 article). Taurine also caused a change in metabolites: decrease in lactate, creatine kinase, phosphorus, inflammatory markers, and improved glycolytic/fat oxidation markers (*n* = 5 articles). Taurine dosing appears to be effective at ~ 1–3 g/day acutely across a span of 6–15 days (1–3 h before an activity) which may improve aerobic performance (TTE), anaerobic performance (strength, power), recovery (DOMS), and a decrease in metabolic markers (creatine kinase, lactate, inorganic phosphate).

**Conclusions:**

Limited and varied findings prohibit definitive conclusions regarding the efficacy of taurine on aerobic and anaerobic performance and metabolic outcomes. There are mixed findings for the effect of taurine consumption on improving recovery from training bouts and/or mitigating muscle damage. The timing of taurine ingestion as well as the type of exercise protocol performed may contribute to the effectiveness of taurine as an ergogenic aid. More investigations are needed to better understand the potential effects of taurine supplementation on aerobic and anaerobic performance, muscle damage, metabolic stress, and recovery.

## Background

Taurine is a sulfur-containing amino acid that can be derived from cysteine metabolism and accounts for 50–60% of the free amino acid pool [6]. Taurine is especially abundant in skeletal muscle [2, 5, 25, 66, 80, 86, 88]. Rich sources of dietary taurine come from the consumption of animal proteins [66]. Taurine plays a beneficial role in diverse metabolic and physiological processes, such as glucose and lipid regulation, energy metabolism, anti-inflammatory modulation, and antioxidant actions [7, 25, 47]. Accordingly, taurine has been used as a potential ergogenic aid to improve athletic performance.

Taurine supplementation often occurs through oral ingestion of capsules or taurine-rich beverages [24, 81]. Notably, plasma concentrations of taurine increase ~ 10 min after ingestion and generally peak (0.03 to 0.06 mmoL) ~ 1 h following ingestion. Importantly, several factors such as taurine ingestion timing, delivery format, and exercise protocol contribute to taurine’s impact on performance. Following this absorption phase, taurine levels return to baseline within 6.5 h (hrs) [24]. However, taurine doses have ranged from 500 mg/d to 10 g/d in published human trials [67]. The purpose of this review is to investigate the literature to date surrounding the effectiveness of taurine supplementation on exercise outcomes: aerobic adaptations and performance, anaerobic (strength and power) performance, muscle soreness, and recovery. For the purpose of this review, we have split the dosages into low (0.5–2 g), moderate (3–5 g), and high (> 5 g). An in-depth explanation of the pharmacokinetics, distribution, and impacts of taurine on metabolism (fat metabolism in particular), and how taurine may impact metabolic stress (inflammation, oxidation, and calcium handling) will be provided first.

### Pharmacokinetics and distribution of Taurine

In humans, one-fourth of bile acids are conjugated with taurine before absorption. Some taurine is converted to isethionate by bacterial or tissue enzymes which are then converted to CO_2_, water, ammonia, or urea. Nevertheless, the vast majority of taurine absorption occurs in the gastrointestinal tract within 1–2.5 h following ingestion [24, 30]. Interestingly, taurine’s bioavailability is improved on an empty stomach [24]. Plasma taurine has been shown to return to baseline concentrations 6–8 h after ingestion [24]. Taurine concentrations are regulated by the kidney via urine excretion; urine excretion of taurine can range from 65 to 250 mg daily [24].

High concentrations of taurine are found in different muscle types. For instance, glycolytic muscle fibers have been found to harbor ~ 1–3 μmol/g of taurine, while oxidative muscle contains ~ 15–20 μmol/g [26]. This exemplifies the point that specific transporters and channels must exist within the muscle membranes for taurine’s import and export [26]. Exercise upregulates the concentration of these transporters and channels to assist in metabolism, ATP formation, ion transport, and signaling and performance adaptations [49]. The entry of taurine into the cell is utilizing a specific sodium-dependent transporter while its release is less well studied; it’s possible via a sodium-independent mechanism [82]. The release is likely due to depolarization of the cell via sodium entry and taurine carriers to the plasma membrane [17]. The mechanical entry of taurine into the cell likely depends on the activation of sodium channels within the muscle cell membrane during exercise.

The skeletal muscle remains the most probable explanation to elucidate the increase in plasma taurine concentration released from the skeletal muscles assayed after exercise. The peak in plasma taurine concentrations occurs immediately at the cessation of exercise [82]. Taurine release could be related to a variety of factors including changes in electrophysical properties of the muscle membrane, sodium concentrations, its co-release with water to maintain plasma volume, and in some undefined way, Ca^2+^ homeostasis. The intensity and speed of exercise are also strongly correlated with high taurine levels; they could play a role in taurine release performed during endurance exercise, possibly indicating its release from oxidative muscle fibers [26, 82]. The increase in taurine release is likely due to taurine’s role in the regulatory mechanism of Ca^2+^ handling within the skeletal and cardiac muscle during contraction, as well as increasing the sensitivity of force-generating myofilaments to calcium [17, 18, 50]. Thus, taurine release and appearance may modulate the concentration of transporters and channels, impacting calcium entry, fat metabolism, and exercise performance.

### Impacts on metabolism

#### Taurine and fat metabolism

Taurine modulates fat metabolism [25, 47, 62]. Acute supplementation of taurine can increase lipolysis and reduce the contribution from glycolytic metabolism, thereby altering the fuel utilization and metabolic efficiency of exercise [14]. This is accomplished, in part, because taurine induces fibroblast growth factors (FGF21, FGF19) and β-klotho concentrations. Fibroblast growth factors (FGFs) are produced in adipose and skeletal muscle tissues and play a role in body energy balance. They act as hormones that decrease blood glucose, insulin, triglycerides, fat mass, carbohydrate metabolism, and may also reduce body weight. Impaired levels of these growth factors could play a role in obesity and cardiovascular diseases [7, 25, 74]. However, taurine may assist with regulating these growth factor levels by increasing fat metabolism and lipolysis through exercise [25]. Exercise increases these growth factors by affecting their gene expressions [74]. Previous literature states that FGF21, FGF19, and β-klotho are implicated in metabolic diseases such as obesity and type 2 diabetes [25]. Importantly, taurine has been shown to reduce these adipose-tissue-derived factors, and by extension, may play a larger role in body composition and adipogenesis. In parallel, an improvement in performance secondary to taurine supplementation would indirectly impact energy expenditure, lipolysis, and likely improve beta-oxidation of fat stores that have been implicated in metabolic diseases and obesity [25]. Nonetheless, few studies have directly assessed the role of taurine on fat mass, and as such our review on this topic is limited.

Furthermore, exercise activates the PI3K/Akt signaling pathway which serves to upregulate and phosphorylate the FGFs via insulin and the contraction of skeletal muscle [74]. The PI3K/Akt signaling pathway is essential for cell survival, metabolism, proliferation, and its implication in human diseases (i.e., cancer, diabetes, cardiovascular) [22]). Since taurine is found in high concentrations in the skeletal muscle, it can potentially play a role in the signaling of the PI3K/Akt pathway during exercise [26, 73]. The importance of taurine serving as a link to this pathway could serve to increase lipolysis and glucose uptake; however, these mechanisms are unknown.

Taurine concentrations in cardiac mitochondria are estimated in high concentrations to be 70 nmoL/mg [26]). It has been suggested that high concentrations of taurine in the cardiac mitochondria can inhibit mitochondrial apoptosis, oxidative and endoplasmic reticulum stress, and serve as a mitochondrial buffer [34]. The acyl-CoA dehydrogenases which control the β-oxidation of fatty acids are shown to have optimal activity with mitochondrial taurine serving as a mitochondrial buffering agent [26]. For example, the rate of β-oxidation of endogenous fatty acids was 31% lower in the taurine-deficient heart in mice compared to the control heart [65]. Effects of taurine deficiency in the heart can lead to diminished and lower rates of the biochemical reactions that occur in the mitochondria during exercise (i.e., citric acid cycle, fatty acid oxidation, and some of the reactions in the urea cycle) [65, 86]. Stabilizing the pH gradient in the mitochondrial matrix by the presence of taurine as a buffer is essential for maintaining the biochemical processes. Taurine has also been demonstrated to be a constituent of modified uridine residues in mitochondrial tRNA [26]. The modified uridine residues in the mitochondria suggest that taurine may play a role in the mitochondrial matrix and be implicated in mitochondrial diseases such as cardiovascular disease. The association of taurine in the mitochondria and exercise can promote an increase in genes relating to mitochondrial respiratory capacity, mitochondrial biogenesis, fat oxidation, and beta-oxidation flux.

Taurine plays a role in fat metabolism, inhibiting oxidative stress by improving mitochondrial function and improving mitochondrial biogenesis [26]. It is suggested that taurine can play a role in the gene expression of mitochondrial biogenesis by upregulating CPT1, PPAR family (PGC-1α PPARα PPARγ), LPL, ACO1, ACO2, HSL, ACOX1, and CD36; genes possibly improving fat metabolism [15, 25, 26]. The presence of taurine in the mitochondria helps ensure the regulation of proton pumping while upregulating these genes. Without the protection of taurine in the mitochondria, this can instigate mitochondrial degradation and dysfunction and potentially metabolic syndrome diseases (i.e., diabetes and obesity,) thus affecting gene expression and FGFs [29, 47]. Also, in adipose tissue, taurine has been established to decrease the number of M1 macrophages, pro-inflammatory cytokines, and increase the number of M2 macrophages, involved in the clearance of free fatty acids and energy expenditure [25]. Taurine can play a role in inhibiting FGFs and inducing PGC-1α gene expression which is involved in the clearance of free fatty acid (FFA), inhibition of lipotoxicity, and thus energy expenditure [20, 25].

Free fatty acids are involved in a variety of metabolic processes (i.e., Krebs cycle, β-oxidation, oxidative phosphorylation, and electron transport chain) which are vital during exercise to upregulate PGC-1α [20, 38]. Since taurine has been proposed to upregulate the mitochondrial PPAR family of genes, this may help increase mitochondrial biogenesis [20, 38]. Since mitochondrial biogenesis plays a role in aerobic capacity during exercise, this adaptation is important in improving cellular metabolism, energy expenditure, and aerobic capacity for an enhancement in sports performance. However, future research needs to investigate the exact mechanisms of taurine’s role in gene expression and mitochondrial biogenesis in aerobic sports. Although inconclusive, taurine may regulate fat metabolism via adipose-tissue-derived growth factors and gene expression; however, this requires more work as well.

#### Aerobic & Anaerobic Metabolism

Due to taurine’s ability to modulate lipid metabolism by stimulating genes and proteins that are responsible for mitochondrial biogenesis and respiratory function, taurine is associated with improvements in aerobic metabolism [15, 25, 26]. Minimal information on how taurine impacts anaerobic metabolism is available; however, previous research has demonstrated taurine may reduce lactate concentrations [14, 39]. While literature is scarce on the impact of taurine on both anaerobic and aerobic metabolism, more information is known about the impact of taurine on lipolysis (mentioned above).

#### Taurine and inflammation in exercise

Taurine could play a vital role in increasing anti-inflammatory markers. Taurine derivatives (taurine chloramine, taurine bromamine, and taurolidine) prevent vascular permeability, which often occurs due to an increase in neutrophil influx and proinflammatory cytokine production, which is increased by inflammatory stimuli such as exercise [42]. Acute exercise leads to an increase in the mobilization of leukocytes and an increase in circulatory inflammatory mediators, such as TNF-α, interleukin-6 (IL-6), interleukin-10 (IL-10), and C-reactive protein (CRP) [37, 53]. However, the rise in IL-6 and CRP concentrations is related to the duration, intensity, and muscle mass involved during exercise which could affect performance adaptations [13, 37, 69]. The damage from exercise generates the inflammatory IL-6 and CRP, which facilitates the influx of inflammatory neutrophils monocytes, lymphocytes, and cells to repair the damage [36, 69]. This injured muscle initiates a rapid and sequential invasion of inflammatory cell mediators that may persist for days or weeks [4, 52, 76]. The anti-inflammatory cytokines serve as mediators to initiate the breakdown of damaged muscle tissues and repair of muscle tissue to promote tissue repair and performance adaptations [11, 43, 76]. There must be a tight equilibrium between pro-inflammatory agents (i.e., IL-6, CRP, and TNF-α,) and anti-inflammatory response to regulate inflammation, repair the skeletal muscle, and promote adaptations and improvements in performance.

Taurine may play a role to attenuate excessive inflammation during recovery periods. Inflammatory markers were unaltered in untrained men with the ingestion of taurine (50 mg/kg/body mass) for 21 days prior to eccentric exercise [11]. There was no change in inflammatory markers (IL-1B, TNF-α, and IL-10) during the recovery period after eccentric exercise in the taurine group compared with the placebo group. This leads to the speculation that taurine may be involved in several other molecular mechanisms of muscle damage repair but may be able to inhibit oxidative stress [11]. However, with inconclusive research, taurine may be beneficial to the balance between the cytokine response and the anti-inflammatory response following exercise [69].

#### Effect on oxidative stress in exercise

With an increase in oxidative stress from exercise, taurine has been proposed to serve as an antioxidant. However, the mechanisms by which taurine acts as an antioxidant have not been clearly defined. Increased production of reactive oxygen species (ROS), such as superoxide and H_2_O_2_ contributes to the disruption and imbalance of Ca^2+^ ion channels leading to impaired cell viability, endothelial stress, inflammation, oxidative stress, related injuries, impaired muscle contractility, fatigue, and diminished performance [27, 54, 70].

The effects of taurine supplementation on oxidative stress biomarkers after eccentric exercise was performed in rats [70]. Taurine was administered as a 1-ml 300 mg/kg/per body weight (BW) day solution in water by gavage, for 15 consecutive days. Starting on the 14th day of supplementation, the animals were submitted to one 90-min downhill run session and a constant velocity of 10 km/hr. Taurine supplementation decreased superoxide radical production, creatine kinase (CK), lipoperoxidation, and carbonylation levels and increased total thiol content in skeletal muscle, but it did not affect antioxidant enzyme activity after eccentric exercise [70]. Taurine affects skeletal muscle contraction by decreasing oxidative stress, in association with decreased superoxide radical production. Future research is warranted to investigate taurine’s antioxidant role after exercise in human subjects.

Taurine is proposed to function as an antioxidant of ROS and promote recovery after exercise. This may promote an improved cellular environment to tolerate exercise oxidative stress [16, 25, 70, 71, 86, 90, 91]. Additionally, hypo-taurine, a taurine precursor, can act as a hydroxyl radical (OH) scavenger and inhibit lipid peroxidation, and prevent iron self-oxidation [16]. An increase in taurine precursors (i.e, cysteine) can serve to increase taurine synthesis and assist in its antioxidant capabilities, decrease the production of superoxide radicals, and maintain skeletal muscle function [61]. Taurine supplementation is shown to decrease the production of superoxide and oxygen-derived radicals, especially in the sites of high production of oxidative stress (i.e., mitochondria), in the skeletal muscle of rats [70].

Taurine improved total thiol (TT) content after exercise in human subjects [12]. Thiols are vital to maintaining the structural enzymatic and transport functions of the cell [70]. However, reversible oxidation of thiols, in the presence of hydrogen peroxide, can lead to oxidative damage from exercise [75]. One capsule of taurine (50 mg/kg/body mass/ day) consumed for a total of 21 days in untrained men, 14 days prior to the eccentric protocol (3 sets until exhaustion of eccentric elbow flexors at 80% of 1RM) and continuing 7-day post-exercise period improved oxidative stress [12]. Taurine supplementation decreased oxidative stress markers in humans, such as lipoperoxidation, carbonylation, and increased total thiol content after exercise in skeletal muscle [12]. However, taurine supplementation did not alter antioxidant enzymes (superoxide dismutase, catalase, and glutathione peroxidase nor inflammatory markers (TNFa, IL-1B, and IL-10) induced by the eccentric protocol during the recovery period. But, taurine lowered creatine kinase levels and improved isometric and concentric strength by day 16 compared to placebo [12]. Taurine supplementation partly attenuated an increase in xylenol orange and carbonyl protein levels (oxidative stress markers) but increased total thiol content [12]. Since this process occurs post-exercise, taurine may be an effective scavenger and decrease the production of superoxide radicals in muscle cells [12, 54, 70]. Therefore, taurine may act to inhibit oxidative stress, associated with decreased superoxide radical production, which may aid in eliciting adaptations and performance for exercise. With little research performed in humans, it is inconclusive to establish the exact mechanisms by which taurine acts as an antioxidant in performance.

#### Taurine and calcium handling in exercise

Taurine is proposed to generate several physiological effects including assisting the sarcoplasmic reticulum (SR) with Ca^2+^ handling in type I and type II muscle fibers [18] and regulation of calcium homeostasis. These actions are related to an increase in calcium-binding protein (calsequestrin1). Calsequestrin1 helps maintain high quantities of calcium in the sarcoplasmic reticulum, promoting higher availability of calcium for cross-bridge formation and muscle contraction [17, 70, 71]. This is accomplished by taurine assisting with calcium release from the SR binding to the myofibrils of the skeletal muscle. However, it remains unclear that taurine has a combination of effects on ion channels, transporter,s, and enzymes, and modulation of intracellular Ca^2+^ levels since research has only been performed in animals [70, 77]. Moreover, taurine is vital for excitation-contraction coupling mechanisms and muscle performance. Taurine may exert metabolic effects via interactions with the muscle membrane and SR which may improve force and power production; unfortunately, this has not been shown to improve exercise performance [63]. Taurine is a well-established inotropic mediator on cardiac muscle fibers and regulator of calcium and excitation-contraction coupling in animals [17]. However, further research is warranted to examine the effects of taurine in humans and its role in SR calcium release in muscle contraction during exercise.

#### Taurine and lactate disappearance

Taurine is shown to increase mitochondrial buffering and lactate disappearance [16]. However, blood lactate level post-exercise remained unaltered after taurine supplementation (1.66 g in a beverage form) in athletes [63]. It remains lacking that taurine results in a decrease in lactate concentrations [14, 51]. This is likely due to a possible interaction between taurine and calcium’s role in mitochondrial buffering [19]. However, the mechanisms of calcium buffering are poorly understood but it could include calcium-binding proteins to assist with intra-mitochondrial buffering mechanisms [19]. The role of taurine’s relationship with calcium mitochondrial buffering is of importance. Taurine may be used to assist mitochondrial buffering capabilities and the preservation of mitochondrial function during exercise. Further research is needed to elucidate changes in lactate disappearance and mitochondrial buffering after taurine supplementation in athletes.

#### Taurine and glucose metabolism

A few studies have shown that taurine is involved in glucose metabolism, but the mechanism is unknown. Taurine supplementation (2% in drinking water) for 30d improved glucose tolerance and insulin sensitivity in mice [60]. Taurine supplemented mice secreted more insulin in response to glucose. The mechanism involved in the higher insulin release resulted in increases in B-cells sensitivity to the cyclic adenosine 3,5-monophosphate (cAMP)/ protein kinase A (PKA) pathways which play a role in insulin secretion and calcium mobilization [60]. Protein Kinase A, specifically, contributes to the first pathway in insulin release which maintains glucose regulation, carbohydrate metabolism, and favors glycogen re-synthesis [60]. Furthermore, in the β-cell, nutrient stimulation leads to depolarization and Ca^2+^ influx to the muscle cell. This stimulates the cAMP pathway and carbohydrate metabolism [28]. However, taurine may modulate the cAMP/PKA pathways to increase insulin secretion, calcium handling, and maintain the concentration of glucose [60]. This is essential for exercise and performance because these pathways promote glycolysis, hypertrophy, muscle recovery, and control glucose levels [59]. Taurine served to regulate the expression of genes required for the glucose-metabolism stimulating insulin secretion and sensitivity [54]. Taurine exerted hypoglycemic effects by enhancing insulin action by facilitating the interaction of insulin with its receptor [54]. Thus, taurine can serve to increase glycogen synthesis, glycolysis, and glucose uptake in the liver of rats and mice. Contrary, the beneficial effects of taurine with glucose metabolism might work by modifying the post-receptor events of insulin action by modulating its signal transduction pathway [48]. Taurine plays a role in improving insulin sensitivity and carbohydrate metabolism in mice; however, there is no research on its role in human glucose metabolism in sports and exercise [60]. The effect of taurine is shown mainly through animal and in vitro studies; to date, there is no evidence that taurine supplementation increases glucose metabolism adaptations in athletes.

## Main text

### Materials and methods

**Fig. 1:**
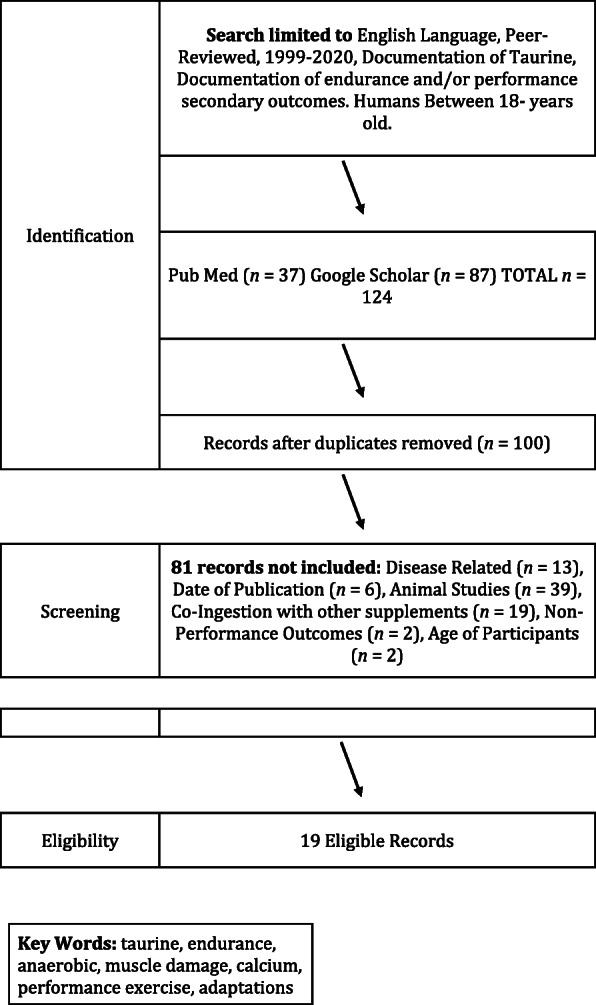
Outline of literature search methodology

All literature that investigated the effect of taurine on aerobic adaptations and performance, anaerobic (strength and power) performance, muscle soreness, and recovery were searched and obtained using the Preferred Reporting Items for Systematic Reviews and Meta-Analyses (PRISMA) statement guidelines, with a pre-determined search strategy [46]. Articles were identified for inclusion via electronic database literature searches. An initial search was conducted using Google Scholar and PubMed, on September 23, 2020. Subsequent searches of Web of Science and PubMed were conducted, using identical search criteria, to capture the most recent publications available (1999–2020). The final search was conducted on September 23, 2020. The full search strategy used for both databases by topic is as follows: ((taurine) AND (aerobic/ endurance); (taurine) AND (submaximal exercise); (taurine) AND muscle damage; (taurine) AND (calcium); (taurine) AND (recovery); (taurine) AND (strength) OR runner* OR cyclist* OR endurance* OR lactate* OR athlete*). Asterisks denote truncation. Additional inclusion criteria included the article was written or available in English, peer-reviewed publication status, and clear information on the administration of taurine. Further criteria included that taurine supplementation was administered in the form of a capsule, powder, or a beverage (1-6 g), and documentation of aerobic or anaerobic performance, muscle damage (e.g., metabolites), muscle soreness, and/or recovery outcomes. The following exclusions were applied to the searches to narrow the scope of the article lists generated: animal studies, non-exercise specific, subjects with epilepsy, atherosclerosis, diabetes, or other health issues. Articles that met inclusion criteria from each database were compiled using Endnote software. Duplicates were removed, and abstracts were pre-screened for the source type. Articles were excluded if taurine was not the primary or only ingredient in a supplement or food source, not published in peer-reviewed journals, if participants were older than 50 years, articles published before 1999, animal studies, or included participants with health issues. After identifying all eligible records, a data matrix was developed and data were extracted on the following variables: study design, sex, athlete type (i.e., sport, training level, age range), recruitment numbers, study length, training protocols, and running/ cycling performance/secondary outcomes. Data from the matrix are presented in Fig. 1. Results were synthesized qualitatively.

## Results – overview

Among the 19 studies included in this review, sex and athlete type were inextricable variables. Seven of the 19 studies examined athletes [2, 3, 14, 41, 63, 78, 81]. One study examined soldiers [35]. Eleven of the studies examined aerobic parameters [2, 3, 14, 23, 35, 40, 45, 51, 63, 81, 91]. Eight of the studies examined anaerobic measures or recovery [12, 41, 44, 55, 56, 78, 79, 83]. Two of the 19 studies examined VO_2_max outcomes in male athletes [81, 91] and two of 19 studies examined women [23, 78]. Eight of the 19 studies examined metabolic outcomes (i.e., blood lactate, amino acids, oxygen, creatine kinase, etc).

Among the examined studies, the type of subjects varied: one recruited endurance-trained cyclists [63], two recruited male swimmers [3, 14]), two recruited a mixed sample of endurance athletes [35, 81], one recruited trained middle-distance runners [2], seven recruited students with no training experience [12, 40, 51, 55, 79, 91], six recruited male recreationally trained individuals [23, 41, 44, 45, 56, 83], one recruited trained women [78], one study recruited both male and female athletes [23], one recruited healthy active volunteers [23]. Ages for study participants ranged from 18 to 46 years [81].

### Aerobic performance and recovery results

Five of the 11 aerobic studies investigated athletes. VO_2_max outcomes (mL/kg/min; *n* = 2 studies) were mixed: one study reported significant increases in VO_2_ [91] and one study reported no difference [35]. An improvement in 3-km time trial performance and relative oxygen consumption was found in one study [2]. An increase in time trial performance was found in untrained males [40, 51, 79, 91], and cyclists [51]. Seven investigations resulted in no improvements in aerobic performance measures [3, 14, 23, 35, 45, 63, 82].

### Anaerobic performance and recovery results

Three studies reported an improvement in anaerobic performance measures [78, 79, 83]. Two of eight anaerobic and recovery studies investigated athletes. An increase in strength and a decrease in muscle soreness was found in three studies [12, 55, 56]). An increase in recovery was found in one study [44]. In fasted noncaffeine users, acute taurine ingestion impairs maximal voluntary muscle power, maximal isokinetic, and isometric peak torque; however, in caffeine-deprived consumers, taurine increased maximal voluntary muscle power [41]. One study with female athletes showed a decrease in heart rate during anaerobic exercise [78].

### Aerobic and anaerobic metabolic markers and stress

A decrease in lactate and anaerobic contribution was found in two studies [14, 51]. A decrease in phosphorus concentration in a time to exhaustion test as noted in a study by Lee and colleagues [40]. Interestingly, two studies reported increases in lipolytic markers and fat oxidation [14, 63]. There was a decrease in creatine kinase blood markers and muscle soreness after 0.5 g for 21 days in male untrained subjects [12]. An improvement in relative oxygen uptake was seen with 1 g of taurine ingestion 2 h prior to a 3 K TT in competitive male runners [2]. However, there was an increase in lactate in a 400 m swim [3] and in an incremental ramp test to volitional exhaustion, followed by 2 min active recovery and 6 × 10 s sprints on a cycle ergometer [78]. In healthy active volunteers, there was no significance in metabolites, but there were notable interaction effects for amino acids, especially improvements in muscle taurine levels during exercise [23].

## Dosage results

### Low dosage - performance

#### Aerobic

Low taurine dosage protocols range from 0.5-2 g. The effect of acute ingestion of 1 g of taurine 2-h prior to a maximal 3-km time trial performance in trained middle-distance runners was examined by Balshaw et al. [2]. To emphasize the importance of supplementation timing, trained soldiers consumed 1 g of taurine supplementation 10–15 min prior to a VO_2_max test [35]. Untrained men, consumed taurine (50 mg/kg) or placebo (3 mg/kg maltodextrin) 2 h prior to a cycling TTE [51]. Male endurance-trained cyclists consumed 1.66 g 1 h before VO_2_max for 90 min followed immediately by a TT [63]. Well-trained male cyclists consumed 1 g of taurine 2 h prior to 3, 4 km cycling time trial performance [81]. Thus, five studies have been conducted to date on low taurine dosing and aerobic performance (Table 1).

**Table 1:** Low taurine supplementation studies to date and outcomes

Author	Participants	Dosage (g)	Timing of Dose	Outcomes
Balshaw et al. [2]	8	1	Placebo or a 1000 mg T (capsule) 2 h ingestion period	↑ 3KTT performance (TA 646.6 ± 52.8 s and PL 658.5 ± 58.2 s) equating to a 1.7% improvement; ↑ Relative oxygen uptake, HR, RPE. No change in blood lactate between conditions
Kammerer et al. [36]	14	1	Four beverages drinking 250 ml of one of the following: one with 80 mg caffeine, one with 1000 mg taurine, one with 80 mg caffeine plus 1000 mg taurine, a commercial energy drink (Red Bull®) or a placebo drink 10–15 min prior to a maximal exercise bout	Combination of caffeine (80 mg) + taurine (1000 mg) in an energy drink prior to exercise results in no effects on cardiorespiratory fitness indices (VO_2_max, HRmax, TTE), strength (RHS and LHS), power (VJ), concentration (Grid) and immediate memory (Digits); No significance of taurine alone on aerobic parameters
Page [52]	11	0.5	Taurine (50 mg kg − 1) or placebo (3 mg kg − 1 maltodextrin) 2 h prior to exercise	↑ TTE by 10% (25.16 min vs. 22.43 min, *p* = 0.040), end sweat rate by 12.7% and ↓ [La] by 16.5%. Core temperature ↓ in the final 10% of the time to exhaustion (38.5 °C vs. 38.1 °C, *p* = 0.049); ↓ RPE
Rutherford et al. [64]	11	1.66	Consumed a noncaloric sweetened beverage with either 1.66 g of T or nothing added (P) 1 h before exercise	No difference in TT performance between any of the 3 trials; Average carbohydrate and fat oxidation rates were unaffected; 16% ↑ (5 g, ~ 84 kJ; *p* < .05) total fat oxidation
Waldron et al. 2018 [80]	9	0.5	High cadence (90 r/min) + taurine (50 mg/kg body mass); high cadence + placebo (3 mg/kg body mass maltodextrin); low cadence (50 r/min) + taurine; low cadence + placebo 1.5 h prior to exercise	↓ HR vs. placebo prior to the ramp test; ↑ blood lactate in taurine conditions; ↑ end-test power in taurine conditions
Waldron et al. 2019 [81]	12	0.5	Participants were allocated to one of four conditions, separated by 72 h: TTE + taurine; TTE + P; 3MAOT + taurine; 3MAOT + placebo 1.5 h prior to exercise	↑ CP (*P* < 0.05) (212 ± 36 W) than baseline (197 ± 40 W) and placebo (193 ± 35 W); Work and power not affected; ↑ TTE 5% > CP increased by 1.7 min after TAU (17.7 min) compared to placebo (16.0 min); ↑ estimated time across all work targets
Ward et al. [83]	11	1	Trials were performed two hours after the consumption of either 1000 mg of TAU or placebo (P)	↓ of TAU on performance; no effect on VO_2_, lactate, pH, or HCO3
Warnock et al. [85]	7	0.5	C (5 mg/kg/ BM), T (50 mg/kg/ BM), C + T (5 mg/kg/ BM + 50 mg/kg/ BM) or P (5, 49 mg/kg BM) in a gelatin capsule 1 h prior to exercise	↑ in anerobic performance compared to P, C or C + T, 63

↑ = improved performance, ↓ = decreased performance, *C* creatine, *T/TAU* taurine, *BM* bodymass, *P* placebo, *mg/kg* milligram per kilogram, *TTE* time to exhaustion, *3KTT* 3-km time trial, *RHS* right-handed grip strength, *LHS* left-handed grip strength, *VJ* vertical jump, *RPE* rate of perceived exertion, *La* lactate, *CP* critical power, *3MAOT* 3-min-all-out-test

#### Anaerobic

A dosage of 0.5 g 1.5 h prior to an exercise bout was recorded in untrained men and trained lacrosse players [78, 79]. Single doses (0.5 g 1.5 h prior to exercise) were equally effective as chronic loading periods in untrained and trained subjects [78, 79] (Table 2). A noticeable decrease in DOMS was found following taurine ingestion (2 g) three times a day, for 2 weeks prior to and 3 days after eccentric elbow flexor exercises [55, 56]. Recreationally trained males consumed 0.5 g prior to three Wingate tests, each separated by 2-min, an hour after ingesting taurine [83]. Thus, three studies have been conducted to date on low taurine dosing and anaerobic performance (Table 1).

**Table 2:** Chronic- dose taurine supplementation studies to date and outcomes

Author	Participants	Dosage (g)	Timing of Dose	Outcomes
Batitucci 2018 [3]	14	3	3 g of pure taurine (capsule) every day in the morning before breakfast, during an eight-week period	No changes in energy expenditure or swimming performance; ↑ in plasma taurine and lactate concentrations
da Silva et al. [12]	21	.5 g·kg/BW/day	One capsule per day for a total of 21 days, beginning 14 days before the eccentric protocol and continuing throughout the 7-day postexercise period	↑ strength levels and thiol total content; ↓ muscle soreness, LAD level, CK activity, and oxidative damage; Antioxidant enzymes and inflammatory markers, and IL-10 were not altered during the recovery period compared with the placebo group
Galloway et al. [23]	7	First part: 1.66 g. Second part: 5 g/day and 6 g/day of placebo	First part: Seven days of oral taurine (1.66 g) supplementation with breakfast and lunch. Second part: cycled for 2 h after 7 days of placebo ingestion (6 g glucose/day) and again following 7 days of T (5 g/day)	No difference in muscle glycogen or other muscle metabolites between conditions; ↑ the appearance of AA following exercise; A 13-fold ↑ increase in plasma taurine concentration; No aerobic effects
Lee et al. [41]	24	4	Subjects were randomly divided into 4 groups (*n* = 6), and given a placebo, taurine (4 g/day), carnitine (4 g/day), or glutamine (4 g/day) tablets for 2 weeks.	↑ TTE (6.9 min or 9.0 min longer) at 75% of VO_2_max; ↓ serum Pi concentration measured at all-out state (14% ↓)
McLeay et al. [45]	10	0.1 g/kg/BW; No more than 10 g	Completed 60 eccentric contractions of the biceps brachii muscle at maximal effort. Following this, participants were supplemented with 0.1 g/kg/BW of either taurine or rice flour in capsules; evening.	Significant ↓ in all performance measures from pre- to 24-h post-eccentric exercise (*p* < 0.001) for both taurine and placebo; No effects in CK levels
Ra et al. [56]	29	2	Participants orally consumed 2 g of placebo (lactose) or taurine powder three times a day after meals for 2 weeks before exercise	↓ DOMS
Ra et al. [57]	36	2	Placebo + placebo [placebo], BCAA + placebo, placebo + taurine, and BCAA + taurine [combined]) and given a combination of 3.2 g BCAA (or placebo) and 2.0 g taurine (or placebo), three times a day, for two weeks prior to and three days after eccentric elbow flexor exercises	↓ exercise-induced DOMS and muscle damage
Zhang et al. [91]	11	6	After the first exercise test, the subjects received supplements of a daily dose of 6 g (2 g three times a day) taurine powder for 7 days prior to the second exercise test. After 7-day of taurine supplementation, an identical exhaustive test procedure was repeated at the same time of day	↓ serum TBARS before exercise (p < 0.05) and resulted in a significantly ↓ DNA migration 24 h after exercise (p < 0.01). Significant ↑ VO_2_max, ↑ exercise TTE, and ↑ maximal workload in test with taurine supplementation (*p* < 0.05); ↑ taurine concentration

↑ = improved performance, ↓ = decreased performance, *T/TAU* taurine, *BW* bodyweight, *P* placebo, *LAD* lactate dehydrogenase, *CK* creatine kinase, *IL-10* interleukin-10, *AA* amino acids, *TTE* time to exhaustion, *Pi* inorganic phosphorus, *DOMS* delayed onset muscle soreness, *TBARS* thiobaribituric-acid reative substance

### Moderate dosage

#### Aerobic

Moderate taurine dosages range from 3 to 5 g. Elite male swimmers ingested 3 g of taurine every day in the morning for 8-weeks prior to 400-m front-crawl submaximal efforts [3]. In a single-blind acute dosing study, subjects cycled for 2 h following a placebo for 7 days (6 g glucose/day) and again taurine (5 g/day) for 7 days [23]. Male untrained college students consumed 4 g in capsule form for 14 days prior to performing a running TTE test [40]. Thus, three studies have been conducted to date on moderate taurine dosing and aerobic performance (Tables 2 and 3).

**Table 3:** Moderate taurine supplementation studies to date and outcomes

Author	Participants	Dosage (g)	Timing of Dose	Outcomes
Batitucci 2018 [3]	14	3	3 g of pure taurine (capsule) every day in the morning before breakfast, during an eight-week period	No changes in energy expenditure or swimming performance; ↑ in plasma taurine and lactate concentrations
da Silva et al. [12]	21	.5 g·kg body mass/day (~ 4)	One capsule per day for a total of 21 days, beginning 14 days before the eccentric protocol and continuing throughout the 7-day postexercise period	↑ strength levels and thiol total content; ↓ muscle soreness, LAD level, CK activity, and oxidative damage; Antioxidant enzymes and inflammatory markers, and IL-10 were not altered during the recovery period compared with the placebo group
Galloway [23]	7	First part: 1.66 g. Second part: 5 g/day and 6 g/day of placebo	First part: Seven days of oral taurine (1.66 g) supplementation with breakfast and lunch. Second part: cycled for 2 h after 7 days of placebo ingestion (6 g glucose/day) and again following 7 days of T (5 g/day)	No difference in muscle glycogen or other muscle metabolites between conditions; ↑ the appearance of amino acids following exercise; A 13-fold ↑ increase in plasma taurine concentration; No aerobic effects
Lee et al. [41]	24	4	Subjects were randomly divided into 4 groups (*n* = 6), and given a placebo, taurine (4 g/day), carnitine (4 g/day), or glutamine (4 g/day) tablets for 2 weeks.	↑ TTE (6.9 min or 9.0 min longer) at 75% of VO_2_max; ↓ serum Pi concentration measured at all-out state (14% ↓)
Lim et al. [42]	14	~ 3	Either cellulose-filled placebo capsules (10 mg/kg body mass; P) or capsules providing 40 mg/kg1 body mass of taurine with 10 mg/kg1 body mass of cellulose (TAU) and drank 250 mL of water one hour prior to testing	In the noncaffeine consumers, taurine resulted in a significant ↓ in maximal voluntary muscle power, ↓ in peak torque, ↓ in first and best power output; taurine ingestion in caffeine-deprived caffeine consumers ↑ maximal voluntary muscle power; no effect on other aspects of contractile performance

↑ = improved performance, ↓ = decreased performance, *T/TAU* taurine, *P* placebo, *LAD* lactate dehydrogenase, *CK* creatine kinase, *IL-10* interleukin-10, *TTE* time to exhaustion, Pi = inorganic phosphate

#### Anaerobic

Untrained men consumed .5 g/kg/body mass/day (~ 4 g) for 21 days before the eccentric protocol [21] (Table 2). Healthy, active volunteers (male and female) consumed mixed low-to-moderate doses of taurine at multiple time periods. Specifically, the acute period was 7 days of oral taurine (1.66 g) supplementation with breakfast and lunch. Power-trained male athletes ingested ~ 3 g in capsule form 1 h prior to performing four isokinetic or three maximal isometric knee extensions with and without taurine [41]. Two studies have been conducted to date on moderate taurine dosing and anaerobic performance (Table 3). See Table 3 for moderate-dose taurine summary.

### High dosage

#### Aerobic

High dosages are > 5 g but not exceeding 10 g. Trained swimmers consumed 6 g 120 min before a 400 m swimming maximal crawl effort. Results from the investigation indicated no improvement in performance but increased plasma glycerol levels [14]. The highest taurine dosage utilized in a human study (myotonic dystrophy patients) was up to 10 g/d for a duration of 6 months [67] (not included in this review, but for context). Male recreationally trained volunteers consumed 6 g 90 min prior to a TTE test [45]. College untrained men consumed 6 g (2 g three times a day) of taurine for 7 days prior to two identical bicycle ergometer exercises until exhaustion [91] (Table 2). Thus, three studies have been conducted to date on high taurine dosing and aerobic performance (Table 4).

**Table 4:** High taurine supplementation studies to date and outcomes

Author	Participants	Dosage (g)	Timing of Dose	Outcomes
De Carvalho et al. [14]	9	6	Athletes received taurine (TAU) or placebo (P) supplementation 120 min before the swimming effort	No effect in swimmer performance; ↑ glycerol plasma levels (8%); ↓ lactic anaerobic system contribution
McLeay et al. [45]	10	0.1 g/ kg/BW (not exceeding 10 g)	Completed 60 eccentric contractions of the biceps brachii muscle at maximal effort. Following this, participants were supplemented with 0.1 g/kg/BW of either taurine or rice flour in capsules; continued to consume the capsules in the morning and evening.	Significant ↓ in all performance measures from pre- to 24-h post-eccentric exercise (*p* < 0.001) for both taurine and placebo; No effects in CK levels
Milioni et al. [46]	17	6	Placebo (6 g dextrose) or taurine (6 g) supplementation), separated by 1 week, 90 min prior to the exhaustive test	No improvements in high-intensity running performance; unclear effect on maximal accumulated oxygen deficit
Ra et al. [56]	29	2	Participants orally consumed 2 g of placebo (lactose) or taurine powder three times a day after meals for 2 weeks before exercise: two sets of maximal eccentric unilateral contractions of the elbow flexor muscle	↓ DOMS
Ra et al. [57]	36	2	Placebo + placebo [placebo], BCAA + placebo, placebo + taurine, and BCAA + taurine [combined]) and given a combination of 3.2 g BCAA (or placebo) and 2 g taurine (or placebo), three times a day, for two weeks prior to and three days after eccentric elbow flexor exercises	↓ exercise-induced DOMS and muscle damage
Zhang et al. [91]	11	6	After the first exercise test, the subjects received supplements of a daily dose of 6 g (2 g three times a day) taurine powder for 7 days prior to the second exercise test. After 7-day of taurine supplementation, an identical exhaustive test procedure was repeated at the same time of day	↓ serum TBARS before exercise (*p* < 0.05) and resulted in a significantly ↓ DNA migration 24 h after exercise (*p* < 0.01). Significant ↑ VO_2_max, ↑ exercise time to exhaustion, and ↑ maximal workload in test with taurine supplementation (*p* < 0.05); ↑ taurine concentration

McLeay [45] dosages ranged from low-to-moderate-to-high; ↑ = improved performance, ↓ = decreased performance, *T/TAU* taurine, *BW* bodyweight, *P* placebo, *CK* creatine kinase, *DOMS* delayed onset muscle soreness, *BCAA* branched chain amino acid, *TBARS* thiobaribituric-acid reative substance

#### Anaerobic

Within the completion of 60 eccentric contractions of the biceps brachii muscle on an isokinetic dynamometer at maximal effort, recreationally trained individuals consumed 0.1 g/kg/BW (not exceeding 10 g) of taurine [44]. Untrained men consumed taurine 2 g (3x a day) for 14 days prior to and 3 days after eccentric elbow extension exercise (from the flexed position at 90° to the fully extended position slowly over 5 s [56]. Recreationally trained men consumed 2 g (3x a day) for 14 days prior to and 3 days after maximal eccentric unilateral contractions of the elbow flexor muscle (each set consisting of 20 contractions) [55]. Accordingly, three studies have been conducted to date on high taurine dosing and anaerobic performance (Table 4).

## Discussion

The discussion will include details about investigations that met the inclusion criteria for this review. Specifically, highlights of individual study methods, dosing, participants will be highlighted surround endurance exercise, anaerobic exercise (i.e., strength/power), recovery (i.e., DOMS), and metabolic markers. Following, conclusions are highlighted and synthesize the research to date as it pertains to the aforementioned specific areas.

### Taurine and endurance exercise

The maintenance of taurine concentration in the muscle tissue might be important for enhanced endurance performance. Very few studies confirm the effect of acute or prolonged taurine ingestion on improving endurance performance in humans [2, 40, 51, 91]. However, trained individuals have higher taurine muscle content compared to their untrained counterparts, indicating a potential role of taurine in human endurance exercise performance [2, 26]. It can be speculated that since trained individuals typically have higher muscle taurine levels, supplemental taurine may be less effective and may have a lower level of enhancement in untrained subjects. Alternatively, higher doses of taurine may be required to achieve performance improvements. However, more research is needed to confirm this assumption.

#### Low-intensity endurance

There were no improvements in endurance performance treated with taurine in a low-intensity exercise protocol [23]. Healthy active men and women ingested 1.66 g oral taurine 3x a day for 7 days. Subjects performed a 2 h cycling bout at ~ 60% peak oxygen consumption (VO_2peak_) after the 7-day ingestion period. There was no difference in muscle taurine content at rest (placebo, 44 *±* 15 μmol/l vs. taurine, 42 *±* 15 μmol/l) or after exercise (placebo, 43 *±* 12 μmol/l vs. taurine, 43 *±* 11 μmol/l). Taurine (1.66 g 3x a day for 7 days) did increase muscle content of amino acids (glutamate, aspartate, asparagine, and lysine) and had no effect on blood metabolites, heart rate, or respiratory responses (glucose, lactate, FFA, VO_2_, RER) to 120 min of exercise at ~ 60 VO_2peak_ [23]. The ingestion of a low dosage of taurine for 7 days, despite large acute changes in plasma following ingestion, does not support performance improvements or changes in carbohydrate metabolism [23].

#### HighHigh-Intensityurance

The acute ingestion of 1 g of taurine (which was equivalent to 2.5 times the maximum daily quantity of taurine intake reported in normal human dietary analysis) 2 h prior to a maximal 3-km time trial performance in trained middle-distance runners significantly enhanced 3-km running performance [2]. Subjects dietary intake 48-h before the performance session was recorded. This investigation was conducted in a simulated endurance performance assessment, more similar to real-world competitive endurance events than previous taurine ingestion studies.

Ten male competitive subjects received 6 g of taurine or placebo 120 min before a front-crawl maximal 400 m swim effort and resulted in no improvement pre-and and post-swim performance [14]. However, it should be noted that plasma glycerol levels (lipolysis) pre and power were 8% higher for the taurine condition. Lactate concentrations were also lower in the taurine condition (11.4 *±* 5.3%) compared to the placebo (14.2 *±* 3.5%) [14]. Swimmer performance was not statistically significant between the placebo and taurine conditions. Since the peak concentration of plasma taurine can be achieved 1.5 to 6 h post-ingestion, the maximal effort of the 400 m swim was performed within the peak concentration period (120 min) and could have promoted an additional effect on lipid metabolism-post exercise [14]. This relationship between taurine’s timing, glycolytic system’s energy contribution, and performance improvement could play a role in lactate disappearance after exercise. Similarly, a 6 g dose of taurine consumed by recreationally trained males 90 min prior to completing a high-intensity exhaustive treadmill running test (110% VO_2_max) failed to show an improvement in endurance performance [45]. Supplementation timing (90 vs 120 min) and exercise protocol design likely play significant roles in taurine’s ability to impact high-intensity endurance performance.

Little research is understood about taurine’s effects on high-intensity repeated sprint performance [83]. Male university team sport players ingested 50 mg/kg dose of taurine supplementation 1 hour prior to three, maximal effort, 30s Wingate anaerobic capacity tests. However, taurine outperformed caffeine (5 mg/kg) and caffeine + taurine (5 mg/kg BM + 50 mg/kg BM) on performance changes. There was an increase in anaerobic performance compared to placebo, caffeine, or caffeine + taurine. Taurine elicited higher peak power, mean power, and mean peak power compared to caffeine + taurine and placebo [83]. Taurine supplementation may increase performance improvements during short, high-intensity events. However, further research is needed to establish this effect and to reveal the underlying mechanisms that explain the current findings.

#### Time to exhaustion and VO2

An increase in running time to exhaustion has been shown to delay fatigue and increase endurance performance. Time to exhaustion running performance was investigated after participants were given a placebo, taurine (4 g/day), carnitine (4 g/day), or glutamine (4 g/day) tablets for 2 weeks [40]. In the taurine condition, the subjects ran 6.9 min longer until exhausted on a treadmill at the intensity of 75% VO_2_max [40]. Serum lactate concentrations measured 1 h after the initiation of the endurance exercise, as well as at an all-out state (immediately following cessation of TTE test) were decreased by taurine, carnitine, and glutamine supplementation. There were no significant changes detected in the lactate concentrations between the three conditions. However, taurine supplementation significantly reduced the serum inorganic phosphorus concentration measured at an all-out state (14% decrease, immediately following cessation of TTE test), assumingly preventing the accumulation in other organelles and continuing cross-bridge formation [1, 87]. Taurine significantly decreased serum ammonia concentration (32% reduction) during the time to exhaustion test [40]. Taurine or carnitine supplementation (4 g/day) improved the time to exhaustion endurance exercise performance and related human fatigue factors.

Higher doses of taurine (4–6 g) in trained and untrained individuals compared to that of typical energy drinks (1–2 g) were investigated [78–80]. Male and female subjects consumed taurine (.5 mg/kg BM in capsule-form) 1.5 h prior to exercise; both groups performed different modalities (time-to-exhaustion trials, time-trials, end-test power output, and critical power output [78, 79]. The ingestion of capsule-form of taurine improved TTE endurance performance and critical power by a small amount, with larger effects found for TTE trials [79, 80].

An improvement in VO_2_max and TTE was seen in untrained men after frequent ingestion of 2 g, 3X a day [91]. Endurance performance could possibly be improved to a similar magnitude after providing 1 g of taurine in a single oral dose or 6 g for up to 2 weeks [2, 51, 91].

#### Mixed aerobic exercise

The consumption of taurine was studied to support the emotional state and aerobic performance in soldiers [35]. Soldiers consumed 250 ml of water with one of the following mixtures: 80 mg caffeine, 1000 mg taurine, 80 mg caffeine plus 1000 mg taurine, a commercial energy drink (Red Bull) or a placebo 10–15 min before a VO_2_max, time to exhaustion, strength (isometric strength), power (vertical jump), concentration (Grid test) and memory test (digits test). There was no significance among the commercial drinks, drinks with different bioactive compounds, and placebo in the various tests performed [35]. However, peak taurine concentrations may not have been attained which could have affected the performance results [24, 30].

Taurine was investigated to evaluate the effects of oxidative stress, aerobic capacity, and maximal workload in untrained males [91]. Subjects performed one VO_2_max test then received 2 g three times a day in a powder for 7 days prior to a second VO_2_max test. Taurine improved VO_2_max (43.7 *±* 4.7 ml/kg/min vs. 46.7 *± 5.3 ml/kg/min), exercise time (18.8 ±* 3.2 ml/kg/min vs. 19.3 ± 3.4 ml/kg/min), and workload (234 ± 65 W vs. 243 ± 67 W) *after 7 days of supplementation. Even though there may have been familiarization between the two tests, taurine concentration levels were positively correlated with changes in time to exhaustion and maximal workload.*

#### Cycling time trial performance

During a prolonged cycling bout in trained athletes, taurine’s ability to increase fat oxidation and performance in a subsequent trial was recorded [63]. Male cyclists utilized a 1 h ingestion period followed by a further 1.5-h sub-maximal 90-min intensity ride before their cycling time trial [63]. Subjects ingested 1.66 g of taurine or a placebo in beverage form. Differences in substrate oxidation between conditions were assessed in the intervening 2.5 h between ingestion and the start of the cycling time trial. Taurine (1.66 g) taken 1 h before 90 min of cycling at ~ 65 VO_2_max resulted in a small, but significant, 16% increase in total whole-body fat oxidation in endurance-trained men but no improvements in 90 min of cycling at 66.5% ± 1.9% VO_2_max [63]. The dosing protocol was based on a previous study [23], and it should be reemphasized that plasma taurine kinetics peak at 90–120 min [24, 30]. However, their results did not explain the exact mechanisms responsible for the increase in total whole-body fat oxidation from taurine alone. Similarly, there was no effect on three, 4 km cycling time trial performance, VO_2_, lactate, pH, or HCO_3_^−^ after consumption of 1 g (2 h prior to exercise bout) of taurine in endurance male athletes [81]. Given the results of the aforementioned investigation on taurine and cycling time trial performance, it can be concluded at this time that taurine supplementation provides no time trial benefits; however, only two investigations have been conducted.

#### Thermoregulation and endurance exercise

Thermal strain (prolonged exercise in high environmental temperatures or humidity) negatively impacts performance [51]. Amino acids have been shown to play a role in thermoregulation and the sweat response within the central nervous system during exercise.

The role of taurine in thermoregulatory control processes and improving time to exhaustion was examined in non-heat acclimated healthy male participants [51]. Taurine supplementation (.5 g) 2 h before a cycling volitional time to exhaustion test in the heat (35 °C, 40% relative humidity) lowered core temperature and improved time to exhaustion by ~ 10%. Subjects cycled at the power output associated with their tested ventilatory threshold until absolute exhaustion. The subject’s core temperature (38.5 °C vs. 38.1 °C) was lower in the final 10% (taurine = 25.16 ± 5.25 min; placebo = 22.43 ± 4.28 min) of the time to exhaustion following taurine supplementation [51]. Taurine supplementation increased time to exhaustion, mean sweat rate (12.7%), decreased RPE and core temperature, in the later stages of exercise, and reduced post-exercise blood lactate concentrations [51]. This is likely due to taurine’s ability to improve thermoregulation, mechanical efficiency, and sweat response [31, 32, 51].

The sweat response from eccrine glands is governed by the thermoregulatory center in the hypothalamus which increases the sympathetic nervous system to increase sweat production [51]. Taurine ingestion appeared to augment the sweat response by increasing the core temperature in the later stages of the bout. This response could elicit taurine’s role as a neuromodulator in the brain and serving as the amino acid gamma-aminobutyric acid (GABA) receptor agonist [32]. Gamma-aminobutyric acid, a brain chemical, is widely distributed in the brain with high concentrations in the hypothalamus [31]. It is considered the majority inhibitory neurotransmitter in most of the brain and also involved with thermoregulation [31]. This brain chemical plays a central role in reciprocal inhibition between the effector pathways controlling thermoregulation [31]. Taurine and GABA are both released from the hypothalamus, the main thermoregulatory center of the brain, suggesting taurine’s importance in temperature regulation [50, 51]. The accumulation of taurine in these central locations presumably reduces core temperature via taurine binding sites or antagonism of GABA receptors. These receptors are established effectors of hypothermia through distinct neural pathways [51]. Higher plasma availability following ingestion of taurine is likely to cross the blood-brain barrier into these central areas, where it can interact with target receptors and prompt thermoregulatory responses. Given taurine’s capacity to cross the blood-brain barrier and elicit changes in time to exhaustion and mean sweat rate in the heat, shows taurine’s implications to assist with thermoregulation in sports performance and exercise. However, with the lack of evidence, more research is needed to elucidate taurine’s role in thermoregulation in sport and exercise.

#### Aerobic capacity

Since taurine has protective redox actions on antioxidation, membrane stabilization, calcium flux, and thermoregulation, taurine was investigated to evaluate the effects of oxidative stress, aerobic capacity, and maximal workload in untrained males [91]. Subjects performed one VO_2_max test then received 2 g taurine three times a day in a powder for 7 days prior to a second VO_2_max test. Taurine improved VO_2_max (43.7 *±* 4.7 ml/kg/min vs. 46.7 *± 5.3* ml/kg/min), exercise time (18.8 ± 3.2 min vs. 19.3 ± 3.4 min), and workload (234 ± 65 W vs. 243 ± 67 W) after 7 days of supplementation. Even though there may have been familiarization between the two tests, taurine concentration levels were positively correlated with changes in time to exhaustion and maximal workload. Since taurine is found in greater concentrations in oxidative muscle fibers, this could suggest taurine could result in endurance exercise improvements [26]. Improvements in endurance performance may be as a result of taurine’s role in calcium flux improving cross-bridge formation, as highlighted in the background of this review [17, 70, 71]*.*

#### Conclusions on endurance

Outcomes related to taurine supplementation and endurance performance are largely mixed [14, 23, 35, 45, 63, 81]. Improvements in endurance performance may be as a result of taurine’s role in calcium flux improving cross-bridge formation [17, 70, 71].

Dosages vary (1 g – 6 g) in improving endurance performance in trained and untrained individuals [14, 45, 79, 91]. Single doses may be equally as effective as chronic loading periods to improve endurance performance without reaching taurine’s upper tolerable limit of 10 g/day [67, 80].

Since taurine is found in greater concentrations in oxidative muscle fibers, this could suggest taurine may improve endurance exercise performance [26]. The enhancing effect of taurine on endurance performance may be a result of subjects utilizing more type I muscle fibers during aerobic events [26]. However, there is little evidence of increasing endurance exercise. Prior taurine ingestion before endurance exercise may attenuate taurine losses from the muscle but it is inconclusive. Future research should investigate the effects of different doses of oral taurine supplementation across participants of varying ages, sex, health, and training status.

### Taurine and muscle soreness

Previous studies have evaluated the effectiveness of branched-chain amino acid (BCAA) supplementation for preventing delayed onset muscle soreness (DOMS) and muscle damage induced by eccentric exercise [56–58, 68, 85]. Although taurine is not a BCAA, taurine could possibly delay muscle soreness by improving satellite cell activation and recovery after a single bout of high intensity, muscle-damaging exercise [10, 78, 79, 83]. Satellite cells are responsible for myofiber development, proliferation, differentiation, and renewal [10, 21]. Since taurine is found in high concentration in the skeletal muscle, after a high bout of exercise or injury, the myofibers are damaged resulting in disruption of the sarcolemma which causes the activation of satellite cells and release of taurine [2, 5, 10, 25, 66, 80, 86, 88].

Delayed onset muscle soreness is one of the symptoms of eccentric exercise-induced muscle damage and can result in prolonged loss of muscle strength, decreased range of motion, muscle swelling, and an increase of muscle proteins in the blood [89]. Muscle damage is characterized as disruption of the membrane by mechanical stress, infiltration of inflammatory cells to the injured tissue, increase in creatine kinase (CK), increased production of inflammatory cytokines, and significant oxidant stress – each factor a potential target with taurine supplementation [56, 84]. As such, taurine is shown to mitigate DOMS. The protection against muscle soreness is of importance for strength improvements and performance adaptations. Taurine supplementation for 21 days (50 mg/kg/day) in male volunteers resulted in reduced CK levels and muscle soreness (DOMS) (lower than those of the placebo group on day 16 and day 18) [12]. Taurine likely helps decrease the amount of muscle soreness during the recovery period. It is possible that the role of taurine lowering CK levels may assist in membrane stabilization and recovery [12].

Branched-chain amino acids and taurine were evaluated to prevent DOMS and muscle damage after eccentric exercise [56]. Thirty-six untrained male subjects consumed 2 g of taurine or a placebo 3X a day (6 g/day) after every meal for 2 weeks prior to exercise and 3 days after eccentric elbow flexor exercises (6 × 5, 90% maximal voluntary contraction (MVC) [56]. A combination of 3.2 g BCAA and 2 g taurine, three times a day, 2 weeks prior to an eccentric exercise (ECC) bout, and 3 days after, attenuated some markers of DOMS (visual analog scale (VAS)) and muscle damage (serum levels of LDH, 8-OHdG, CK) levels induced by high-intensity eccentric exercise (ECC). However, there was no effect of prolonged taurine use, without the combination of BCAA, on soreness or markers of muscle damage caused by eccentric elbow flexion exercise in untrained men [56]. It appears that the combination of BCAA and taurine is essential to mitigate muscle soreness effects and muscle damage induced by high-intensity ECC.

In a follow-up study, recreational trained subjects used an identical taurine supplementation (2 g of taurine, independent BCAA, or a placebo powder 3x a day after meals for 2 weeks before ECC elbow flexion exercise (2 × 20, MVC) [55]. Supplementation was continued until the third day after exercise. Muscle soreness during a 4-day recovery period after exercise was attenuated in the participants who consumed taurine [55]. Multiple ingestions of taurine supplementation throughout the day significantly reduced the severity of DOMS induced by ECC.

Similarly, taurine (0.1 g) over 3 days following eccentric exercise attenuated the rise in serum creatine kinase and improved performance recovery in recreationally trained males [44]. Subjects performed 60 eccentric contractions of the biceps brachii at maximal effort. Following the exercise bout, participants were supplemented with 0.1 g/kg/bodyweight of either taurine or rice flour in capsules, morning and evening. Neither treatment group fully recovered force output by 72 h over time or between treatments. However, force recovery significantly increased toward pre-values at 48 h with taurine compared to placebo [44]. Taurine may expedite the recovery of eccentric force. The source of the increased taurine plasma content after exercise requires further exploration as this may reflect muscle soreness and force output. Taurine supplementation shows promise as a nutritional adjunct to reduce symptoms of exercise-induced does, though more information is needed to determine optimal dosing patterns.

### Taurine and strength and power

Eccentric actions are characterized by a high force output which is performed in pre-season sports to increase strength and power in the later seasons [8, 9]. Since taurine assists the SR with Ca^2+^ release, this increases the sensitivity of force-generating myofilaments, in both skeletal muscle and cardiac tissue [17, 70], increasing muscle force [64] and thus improving performance outcomes. Even though taurine’s role in endurance performance is more conclusive, research is limited in taurine’s role to increase strength and power. However, the degree of muscle soreness may affect strength improvements and force outputs; taurine may be able to modulate these effects.

Improvements in strength and power with taurine supplementation were notable [12, 41, 78, 79, 83]. The degree of muscle soreness may affect strength improvements and force outputs. The effects of taurine (0.5 g/kg/body mass/day for 21 days) improved concentric and isometric strength during the 48 h after elbow flexion exercise (3 × 11–15, 80% 1RM) in male students [12]. Taurine effectively enhances concentric and isometric strength during the recovery period after exercise. For athletes who perform subsequent training bouts per week during a training season, taurine may be beneficial to improve strength. Increasing strength is important to increase power output [72]. The ability of taurine to affect muscle function and power output in caffeine and caffeine-deprived users were examined [41]. Trained male athletes (who were separated into caffeine and non-caffeine users) ingested a placebo (10 mg/kg body mass) or capsules (40 mg/kg/ body mass of taurine) with 250 mL of water 1 hour prior to four isokinetic or three maximal isometric knee extensions. Taurine ingestion did not affect maximal voluntary muscle power, maximal isokinetic, and isometric peak torque in noncaffeine consumers, whereas taurine ingestion in caffeine-deprived, caffeine consumers improved maximal voluntary muscle power (isokinetic peak torque, isometric peak torque, and isokinetic power). There was no effect on other aspects of contractile performance [41]. Thus, taurine can play a role in isokinetic peak torque, isometric peak torque, and isokinetic power in caffeine-deprived users. This study has important implications for habitual caffeine users wishing to ingest taurine.

Most of the available studies available have focused on taurine’s ability to affect power output in men. However, to our knowledge, one study investigated women’s power output [78]. Female university lacrosse players ingested taurine (50 mg/kg body mass) + maltodextrin (3 g/kg body mass) or placebo (.3 g/kg body mass maltodextrin) 1.5 h prior to a cycling ramp test fixed in the isokinetic mode (90 or 50 cadences) until exhaustion. There was an effect of higher end-test power with taurine compared to placebo. There was no difference in ramp exercise or sprint performance, but taurine ingestion is responsible for an increase in ramp exercise and end-test power output [78]. Although the reasons were unclear, taurine likely plays a role in skeletal muscle contraction function and power output. Females competing in short, aerobically based cycling, may benefit from taurine to produce a greater end-test power output as fatigue starts to increase. In a subsequent study, critical power in men was analyzed [79]. Critical power represents the highest power output that can be maintained for a duration of time, without a continuous rise in VO_2_ and blood lactate concentrations or reductions in phosphocreatine stores [79]. Critical power can be used to assess training status, performance improvements, or the power-time relationship [33]. Taurine is speculated to alter the power-time relationship. Untrained men consumed a taurine capsule (0.5 g/kg/BW) or a placebo (0.3 g/kg/BW) 1.5 h prior to a 3-min all-out test (3MAOT) or a TTE test at a fixed external power output 5% greater than their baseline critical power 3MAOT. There was an improvement during the 3MAOT, peak power, and TTE with taurine supplementation [79]. Taurine could improve TTE and critical power outputs in untrained men. However, the effects of taurine were more effectively demonstrated in the TTE test. This is likely due to a greater concentration of taurine in oxidative muscle fibers [26]. Acute taurine supplementation is shown to increase exercise tolerance and critical power output in men, and end-test power out in women. However, to our knowledge, no peer-reviewed literature exists on taurine’s independent role in skeletal muscle hypertrophy. To date, the experimental evidence on fiber cross-sectional area or muscle mass is lacking. Since taurine is known to increase strength and power as well as improve muscle repair from injury, we can only theorize that taurine must play a role in hypertrophy. The relationship between taurine supplementation and skeletal muscle hypertrophy warrants further investigation.

### Dietary protein intake

Since taurine is found primarily in animal products [66], concentration levels of taurine via the diet can potentially confound taurine supplementation. As such, it is vital to account for a subject’s baseline taurine levels prior to supplementing with additional taurine, as drastically different levels could confound results. It can be assumed that individuals who eat a higher protein diet will likely have higher taurine and amino acid levels in the muscle. The use of these amino acids is important for such pathways as cAMP/PKA [60] and thermoregulation [31]. Thus, if there is a higher concentration of taurine, it may improve the function of these pathways and ultimately sport and exercise performance.

## Conclusions

The majority of the current literature that is available supports that taurine may serve as an anti-inflammatory agent, antioxidant, modulator of ion flux, assist with the control of Ca^2+^ homeostasis, and membrane stabilization effects; however, this literature has primarily been conducted in animal models. This is the first review to examine the impact and application of taurine supplementation on aerobic or anaerobic performance, muscle damage and soreness, and recovery in humans. Taurine has been proposed as an ergogenic supplement in exercise performance due to its abundance in human skeletal muscle and its role in a variety of physiological functions, including energy metabolism, and oxidative stress and inflammation regulation [5, 25, 64, 66, 86, 88]. A combination of factors likely explains taurine’s ‘small’ effects of aerobic and anaerobic performance, muscle soreness, and recovery possibly due to taurine’s bioavailability with timing and dosage.

### Form of Taurine / peak bioavailability

- To date, it appears that capsule form consumption may be the most beneficial, consumed in the morning or afternoon 10–15 min-to-2 h prior to exercise. Taurine investigations to date support this literature synthesized conclusion [2, 41, 51, 78, 79, 83].
  - Beverage forms, however, consumed 10 min – 1 h prior to exercise is inconclusive and future research is warranted to investigate taurine in a beverage form in sports and exercise.
- Since it is an amino acid, a plethora of research [17] has shown that taurine consumption is not harmful when ingested regularly, including training and non-training days. Thus, it is safe to consume on different training days or seasons.
- Taurine needs to attain its peak plasma concentration (0.03 to 0.06 mmoL), which has been demonstrated 10 min – 1 h prior to exercise (generally peaks ~ 1 h following taurine ingestion), providing the greatest bioavailability and potential to improve performance outcomes. Factors such as taurine ingestion timing and exercise protocol may contribute to whether or not taurine enhances performance.

### Small dosage (0.5-2 g)

- The majority of investigations to date indicate smaller dosages are not very ergogenic when it comes to enhancing exercise performance.
- Only three studies performed with athletes at low dosages show performance improvements [2, 41, 78].

### Moderate dosage (3-5 g)

- Mixed results to date. There is little (strength, power, TTE) to no improvement in performance (aerobic) moderate taurine doses.

### High dosage (> 5 g)

- It is unclear whether high doses short or long term improve performance with trained and untrained subjects.
- High doses of taurine are not linked to adverse health effects, such as high blood pressure. As such, taurine supplementation could be explored as a safe and effective supplement [24].

### Chronic dosage

- Chronic supplementation with taurine (> 7 days) in untrained individuals reveal more consistent results [12, 40, 44, 55, 91]

### Dosage conclusion

- 1–3 g/day of taurine administered 60–120 min before the activity, with the same amount given as a chronic dose periodically throughout the day for over 6–21 days has been effective
- Performance improvements can be achieved without a chronic dose period.
- However, long-reimplementation in untrained or recreationally trained individuals resulted in more promising improvements in performance than trained individuals.
- It is recommended to consume taurine 60 min-2 h prior to exercise for peak bioavailability

Thus, the dose, duration, ingestion-time period, exercise protocol, and training status of an individual likely contribute to the effectiveness of taurine to improve performance. Various dosages and duration of taurine make it challenging to elucidate the proper amount for exercise and thus make it inconclusive.

### Training status of individuals

- The population that would most benefit from this supplement remains unclear, given the range of subjects (trained, untrained, males, and females) and duration of consumption
- It is important to consider the impact of type, level, and duration of training, genetics, recovery strategy, and sex of athletes compared to untrained individuals moderating the influence of taurine consumption on performance outcomes.

### Taurine and performance

Approximately 68% of the studies we included in this review reported improvements to a variety of performance variables. While these statistics are encouraging, the study designs, subject population, and experimental outcomes are too variable to fully support taurine’s efficacy as an ergogenic aid. However, taurine has been implicated in a variety of physiological, biochemical, and metabolic processes and thus more research on taurine and performance s certainly warranted.

There is mixed research that taurine improves endurance, strength, and power, and expedites the recovery of DOMS or markers of muscle damage. However, there is an increase in endurance performance improvements compared to anaerobic outcomes (strength and power). The greater concentration of taurine in oxidative muscle fibers may support taurine’s role in endurance events. The mechanisms that elucidate how taurine affects human endurance performance and the appropriate doses for the specific performance outcomes are still unclear. While more research is continually developed regarding the effects of taurine in sports and exercise, the consensus of these outcomes is unclear regarding’s taurine’s role in aerobic and anaerobic performance, muscle damage and soreness, and recovery.

## Data Availability

Access to all data cited in this review paper have been provided in the references list.

## Abbreviations

AA: Amino acid
ACO1: Aconitase-1
ACO2: Aconitase-2
ACOX1: Peroxisomal straight-chained acyl-CoA oxidase
Akt: Protein kinase B
BCAA: Branch-chained amino acid
BW: Body weight
Ca: Calcium
cAMP: Cyclic adenosine monophosphate
CD36: Cluster of differentiation 36
CK: Creatine kinase
CPT1: Carnitine palmitoyltransferase-1
CRP: C-reactive protein
DOMS: Delayed onset muscle soreness
ECC: Eccentric
FFA: Free fatty acid
FGF: Fibroblast growth factor
GABA: Gamma-aminobutyric acid
HSL: Hormone-sensitive lipase
IL: Interleukin
La: Lactate
LAD: Lactate dehydrogenase
LHS: Left hand grip strength
LPL: Lipoprotein lipase
PGC1α: Peroxisome proliferator-activated receptor-gamma coactivator 1
Pi: Inorganic phosphate
PI3K: Phosphoinositide 3-kinase
PKA: Protein kinase A
PPAR: Peroxisome proliferator-activated receptor
PRISMA: Preferred reporting items for systemic reviews and meta-analyses
RER: Respiratory exchange ratio
RHS: Right hand grip strength
RM: Repetition maximum
RPE: Rate of perceived exertion
ROS: Reactive oxygen species
SR: Sarcoplasmic reticulum
Tau: Taurine
TBARS: Thiobaribituric acid reactive substance
TNF: Tumor necrosis factor
TT: Total thiol
TTE: Time to exhaustion
3MAOT: 3-min all-out test

## References

[1] Allen D, Westerblad H (2001). Role of phosphate and calcium stores in muscle fatigue. J Physiol.

[2] Balshaw TG, Bampouras TM, Barry TJ, Sparks SA (2013). The effect of acute taurine ingestion on 3-km running performance in trained middle-distance runners. Amino Acids.

[3] Batitucci G, et al. Effects of taurine supplementation in elite swimmers performance. Motriz: Revista de Educação Física. 2018;24(1):1-7.

[4] Bessa AL, Oliveira VN, Agostini GG, Oliveira RJ, Oliveira AC, White GE (2016). Exercise intensity and recovery: biomarkers of injury, inflammation, and oxidative stress. J Strength Condition Res.

[5] Bongiovanni T, et al. Nutritional interventions for reducing the signs and symptoms of exercise-induced muscle damage and accelerate recovery in athletes: current knowledge, practical application, and future perspectives. Eur J Appl Physiol. 2020;120(9):1–32. 10.1007/s00421-020-04432-3 32661771

[6] Brosnan JT, Brosnan ME (2006). The sulfur-containing amino acids: an overview. J Nutr.

[7] Carvalho MB (2020). Taurine supplementation increases post-exercise lipid oxidation at moderate intensity in fasted healthy males. Nutrients.

[8] Cook CJ, Beaven CM, Kilduff LP (2013). Three weeks of eccentric training combined with overspeed exercises enhances power and running speed performance gains in trained athletes. J Strength Condition Res.

[9] Cowell JF, Cronin J, Brughelli M (2012). Eccentric muscle actions and how the strength and conditioning specialist might use them for a variety of purposes. Strength Condition J.

[10] Crameri RM, Langberg H, Magnusson P, Jensen CH, Schrøder HD, Olesen JL, Suetta C, Teisner B, Kjaer M (2004). Changes in satellite cells in human skeletal muscle after a single bout of high intensity exercise. J Physiol.

[11] da Silva JCG, Silva KF, Domingos-Gomes JR, Batista GR, da Silva Freitas ED, Torres VBC, do Socorro Cirilo-Sousa M (2019). Aerobic exercise with blood flow restriction affects mood state in a similar fashion to high intensity interval exercise. Physiol Behav.

[12] da Silva LA, Tromm CB, Bom KF, Mariano I, Pozzi B, da Rosa GL, Tuon T, da Luz G, Vuolo F, Petronilho F, Cassiano W, de Souza CT, Pinho RA (2014). Effects of taurine supplementation following eccentric exercise in young adults. Appl Physiol Nutr Metab.

[13] Danese E, Tarperi C, Salvagno GL, Guzzo A, Sanchis-Gomar F, Festa L (2018). Sympatho-adrenergic activation by endurance exercise: effect on metanephrines spillover and its role in predicting athlete’s performance. Oncotarget.

[14] De Carvalho FG (2018). Taurine supplementation can increase lipolysis and affect the contribution of energy systems during front crawl maximal effort. Amino Acids.

[15] De Carvalho FG, et al. Taurine supplementation associated with exercise increases mitochondrial activity and fatty acid oxidation gene expression in the subcutaneous white adipose tissue of obese women. Clin Nutr. 2020;40(4):2180-7. 10.1016/j.clnu.2020.09.044 33051044

[16] De Carvalho FG (2017). Taurine: a potential ergogenic aid for preventing muscle damage and protein catabolism and decreasing oxidative stress produced by endurance exercise. Front Physiol.

[17] De Luca A, Pierno S, Camerino DC (2015). Taurine: the appeal of a safe amino acid for skeletal muscle disorders. J Transl Med.

[18] Dutka TL, Lamboley CR, Murphy RM, Lamb GD (2014). Acute effects of taurine on sarcoplasmic reticulum Ca2+ accumulation and contractility in human type I and type II skeletal muscle fibers. J Appl Physiol.

[19] El Idrissi A (2008). Taurine increases mitochondrial buffering of calcium: role in neuroprotection. Amino Acids.

[20] Elżbieta S, Agnieszka M, Adrian C (2017). The implication of PGC-1α on fatty acid transport across plasma and mitochondrial membranes in the insulin sensitive tissues. Front Physiol.

[21] Fernyhough ME, Bucci LR, Feliciano J, Dodson MV (2010). The effect of nutritional supplements on muscle-derived stem cells in vitro. Int J Stem Cells.

[22] Fruman DA, Chiu H, Hopkins BD, Bagrodia S, Cantley LC, Abraham RT (2017). The PI3K pathway in human disease. Cell.

[23] Galloway SD (2008). Seven days of oral taurine supplementation does not increase muscle taurine content or alter substrate metabolism during prolonged exercise in humans. J Appl Physiol.

[24] Ghandforoush-Sattari M, Mashayekhi S, Krishna CV, Thompson JP, Routledge PA (2010). Pharmacokinetics of Oral Taurine in Healthy Volunteers. J Amino Acids.

[25] Haidari F, Asadi M, Ahmadi-Angali K (2019). Evaluation of the effect of oral taurine supplementation on fasting levels of fibroblast growth factors, β-Klotho co-receptor, some biochemical indices and body composition in obese women on a weight-loss diet: a study protocol for a double-blind, randomized controlled trial. Trials.

[26] Hansen S, Andersen M, Cornett C, Gradinaru R, Grunnet N (2010). A role for taurine in mitochondrial function. J Biomed Sci.

[27] He F (2016). Redox mechanism of reactive oxygen species in exercise. Front Physiol.

[28] Hill-Eubanks DC, Werner ME, Heppner TJ, Nelson MT (2011). Calcium signaling in smooth muscle. Cold Spring Harb Perspect Biol.

[29] Imae M, Asano T, Murakami S (2014). Potential role of taurine in the prevention of diabetes and metabolic syndrome. Amino Acids.

[30] Information, NCfB (2020). Compound Summary for CID 1123.

[31] Ishiwata T, Saito T, Hasegawa H, Yazawa T, Kotani Y, Otokawa M, Aihara Y (2005). Changes of body temperature and thermoregulatory responses of freely moving rats during GABAergic pharmacological stimulation to the preoptic area and anterior hypothalamus in several ambient temperatures. Brain Res.

[32] Jia F, Yue M, Chandra D, Keramidas A, Goldstein PA, Homanics GE, Harrison NL (2008). Taurine is a potent activator of extrasynaptic GABAA receptors in the thalamus. J Neurosci.

[33] Jones AM, Vanhatalo A (2017). The ‘critical power’concept: applications to sports performance with a focus on intermittent high-intensity exercise. Sports Med.

[34] Jong CJ (2017). Role of mitochondria and endoplasmic reticulum in taurine-deficiency-mediated apoptosis. Nutrients.

[35] Kammerer M (2014). Effects of energy drink major bioactive compounds on the performance of young adults in fitness and cognitive tests: a randomized controlled trial. J Int Soc Sports Nutr.

[36] Kawamura T, Muraoka I (2018). Exercise-induced oxidative stress and the effects of antioxidant intake from a physiological viewpoint. Antioxidants.

[37] Khazaei M (2012). Chronic low-grade inflammation after exercise: controversies. Iran J Basic Med Sci.

[38] Konopka AR, Suer MK, Wolff CA, Harber MP (2014). Markers of human skeletal muscle mitochondrial biogenesis and quality control: effects of age and aerobic exercise training. J Gerontol A Biol Sci Med Sci.

[39] Kowsari E (2018). The effect of short-term taurine amino acid supplement on neuromuscular fatigue, serum lactate level and choice reaction time after maximal athletic performance. J Res Med Dent Sci.

[40] Lee H, Paik I, Park T (2003). Effects of dietary supplementation of taurine, carnitine or glutamine on endurance exercise performance and fatigue parameters in athletes. Korean J Nutr.

[41] Lim ZX (2018). The effect of acute taurine ingestion on human maximal voluntary muscle contraction. Med Sci Sports Exerc.

[42] Marcinkiewicz J, Kurnyta M, Biedroń R, Bobek M, Kontny E, Maśliński W (2006). Anti-inflammatory effects of taurine derivatives (taurine chloramine, taurine bromamine, and taurolidine) are mediated by different mechanisms. In Taurine.

[43] Maria Carmen G-C, Viña J, Ji LL (2016). Role of Redox Signaling and Inflammation in Skeletal Muscle Adaptations to Training. Antioxidants.

[44] McLeay Y, Stannard S, Barnes M (2017). The effect of taurine on the recovery from eccentric exercise-induced muscle damage in males. Antioxidants.

[45] Milioni F, Malta ES, Rocha LGSA, Mesquita CAA, de Freitas EC, Zagatto AM (2016). Acute administration of high doses of taurine does not substantially improve high-intensity running performance and the effect on maximal accumulated oxygen deficit is unclear. Appl Physiol Nutr Metab.

[46] Moher D (2015). Preferred reporting items for systematic review and meta-analysis protocols (PRISMA-P) 2015 statement. Syst Rev.

[47] Murakami S (2015). Role of taurine in the pathogenesis of obesity. Mol Nutr Food Res.

[48] Nandhini ATA, Anuradha CV (2002). Taurine modulates kallikrein activity and glucose metabolism in insulin resistant rats.

[49] O’Rourke B (2007). Mitochondrial ion channels. Annu Rev Physiol.

[50] Oja SS, Saransaari P. Pharmacology of taurine. Proc West Pharmacol Soc. 1998;50:95-7. 18605222

[51] Page LK, Jeffries O, Waldron M (2019). Acute taurine supplementation enhances thermoregulation and endurance cycling performance in the heat. Eur J Sport Sci.

[52] Pahwa R, Jialal I (2019). Chronic inflammation.

[53] Pournot H (2011). Time-course of changes in inflammatory response after whole-body Cryotherapy multi exposures following severe exercise. PLoS One.

[54] Puerta CD (2010). Taurine and glucose metabolism: a review Taurina y metabolismo de la glucosa: una revisión. Nutrición Hospitalaria.

[55] Ra S-G, et al. Taurine supplementation reduces eccentric exercise-induced delayed onset muscle soreness in young men, in Taurine 9. Cham: Springer; 2015. p. 765–72. 10.1007/978-3-319-15126-7_61 25833543

[56] Ra S-G (2013). Combined effect of branched-chain amino acids and taurine supplementation on delayed onset muscle soreness and muscle damage in high-intensity eccentric exercise. J Int Soc Sports Nutr.

[57] Ra SG, Miyazaki T, Kojima R, Komine S, Ishikura K, Kawanaka K (2018). Effect of BCAA supplement timing on exercise-induced muscle soreness and damage: a pilot placebo-controlled double-blind study. J Sports Med Phys Fitness.

[58] Rahimi MH, Shab-Bidar S, Mollahosseini M, Djafarian K (2017). Branched-chain amino acid supplementation and exercise-induced muscle damage in exercise recovery: a meta-analysis of randomized clinical trials. Nutrition.

[59] Ravnskjaer K, Madiraju A, Montminy M. Role of the cAMP pathway in glucose and lipid metabolism. Metabo Contr. 2015:29–49. 10.1007/164_2015_32. 10.1007/164_2015_32 26721678

[60] Ribeiro RA, Vanzela EC, Oliveira CAM, Bonfleur ML, Boschero AC, Carneiro EM (2010). Taurine supplementation: involvement of cholinergic/phospholipase C and protein kinase a pathways in potentiation of insulin secretion and Ca 2+ handling in mouse pancreatic islets. Br J Nutr.

[61] Ripps H, Shen W (2012). Taurine: a “very essential” amino acid.

[62] Rosa FT, Freitas EC, Deminice R, Jordão AA, Marchini JS (2014). Oxidative stress and inflammation in obesity after taurine supplementation: a double-blind, placebo-controlled study. Eur J Nutr.

[63] Rutherford JA, Spriet LL, Stellingwerff T (2010). The effect of acute taurine ingestion on endurance performance and metabolism in well-trained cyclists. Int J Sport Nutr Exerc Metab.

[64] Schaffer SW, Ju Jong C, KC R, Azuma J (2010). Physiological roles of taurine in heart and muscle. J Biomed Sci.

[65] Schaffer SW, Shimada-Takaura K, Jong CJ, Ito T, Takahashi K (2016). Impaired energy metabolism of the taurine-deficient heart. Amino Acids.

[66] Seidel U, Huebbe P, Rimbach G (2019). Taurine: a regulator of cellular redox homeostasis and skeletal muscle function. Mol Nutr Food Res.

[67] Shao A, Hathcock JN (2008). Risk assessment for the amino acids taurine, L-glutamine and L-arginine. Regul Toxicol Pharmacol.

[68] Shimomura Y, Inaguma A, Watanabe S, Yamamoto Y, Muramatsu Y, Bajotto G, Sato J, Shimomura N, Kobayashi H, Mawatari K (2010). Branched-chain amino acid supplementation before squat exercise and delayed-onset muscle soreness. Int J Sport Nutr Exerc Metab.

[69] Shirvani H, Nikbakht H, Ebrahim Kh GA (2012). The effects of soccer specific exercise and Taurine supplementation on serum cytokine response in male elite soccer players. Ann Biol Res.

[70] Silva L, Silveira PC, Ronsani MM, Souza PS, Scheffer D, Vieira LC (2011). Taurine supplementation decreases oxidative stress in skeletal muscle after eccentric exercise. Cell Biochem Funct.

[71] Spriet LL, Whitfield J (2015). Taurine and skeletal muscle function. Curr Opin Clin Nutr Metab Care.

[72] Stone MH, O'Bryant HS, McCoy L, Coglianese R, Lehmkuhl MARK, Schilling B (2003). Power and maximum strength relationships during performance of dynamic and static weighted jumps. J Strength Condition Res.

[73] Sun G, Wang X, Li T, Qu S, Sun J (2018). Taurine attenuates acrylamide-induced apoptosis via a PI3K/AKT-dependent manner. Hum Exp Toxicol.

[74] Tanimura Y (2016). Acute exercise increases fibroblast growth factor 21 in metabolic organs and circulation. Physiol Rep.

[75] Terrill JR, Duong MN, Turner R, le Guiner C, Boyatzis A, Kettle AJ, Grounds MD, Arthur PG (2016). Levels of inflammation and oxidative stress, and a role for taurine in dystropathology of the Golden retriever muscular dystrophy dog model for Duchenne muscular dystrophy. Redox Biol.

[76] Tidball JG (2005). Inflammatory processes in muscle injury and repair. Am J Phys Regul Integr Comp Phys.

[77] Vettorazzi JF, Ribeiro RA, Santos-Silva JC, Borck PC, Batista TM, Nardelli TR, Boschero AC, Carneiro EM (2014). Taurine supplementation increases K ATP channel protein content, improving Ca 2+ handling and insulin secretion in islets from malnourished mice fed on a high-fat diet. Amino Acids.

[78] Waldron M, Knight F, Tallent J, Patterson S, Jeffries O (2018). The effects of taurine on repeat sprint cycling after low or high cadence exhaustive exercise in females. Amino Acids.

[79] Waldron M, Patterson SD, Jeffries O (2019). Oral taurine improves critical power and severe-intensity exercise tolerance. Amino Acids.

[80] Waldron M, Patterson SD, Tallent J, Jeffries O (2018). The effects of an oral taurine dose and supplementation period on endurance exercise performance in humans: a meta-analysis. Sports Med.

[81] Ward R, Bridge CA, McNaughton LR, Sparks SA (2016). The effect of acute taurine ingestion on 4-km time trial performance in trained cyclists. Amino Acids.

[82] Ward RJ, Francaux M, Cuisinier C, Sturbois X, de Witte P (1999). Changes in plasma taurine levels after different endurance events. Amino Acids.

[83] Warnock R, Jeffries O, Patterson S, Waldron M (2017). The effects of caffeine, taurine, or caffeine-taurine coingestion on repeat-sprint cycling performance and physiological responses. Int J Sports Physiol Perform.

[84] Warren GL, Lowe DA, Armstrong RB (1999). Measurement tools used in the study of eccentric contraction-induced injury. Sports Med.

[85] Watanabe S (2007). Effects of branched-chain amino acid (BCAA) supplementation before and after exercise on delayed-onset muscle soreness (DOMS) and fatigue. FASEB J.

[86] Wen C, Li F, Zhang L, Duan Y, Guo Q, Wang W, He S, Li J, Yin Y (2019). Taurine is involved in energy metabolism in muscles, adipose tissue, and the liver. Mol Nutr Food Res.

[87] Westerblad H, Allen DG, Lannergren J (2002). Muscle fatigue: lactic acid or inorganic phosphate the major cause?. Physiology.

[88] Wu G. Important roles of dietary taurine, creatine, carnosine, anserine and 4-hydroxyproline in human nutrition and health. Amino Acids. 2020;52(3):1–32. 10.1007/s00726-020-02823-6 PMC7088015 32072297

[89] Yu JG, Malm C, Thornell LE (2002). Eccentric contractions leading to DOMS do not cause loss of desmin nor fibre necrosis in human muscle. Histochem Cell Biol.

[90] Zembron-Lacny A, Szyszka K, Szygula Z. Effect of cysteine derivatives administration in healthy men exposed to intense resistance exercise by evaluation of pro-antioxidant ratio. J Physiol Sci. 2007;57(6):343-8. 10.2170/physiolsci.RP009307 17999779

[91] Zhang M, Izumi I, Kagamimori S, Sokejima S, Yamagami T, Liu Z, Qi B (2004). Role of taurine supplementation to prevent exercise-induced oxidative stress in healthy young men. Amino Acids.

